# Clinical Outcomes and Retreatment Pathways Following Intradetrusor OnabotulinumtoxinA in Women with Refractory Idiopathic Overactive Bladder

**DOI:** 10.3390/jcm15186977

**Published:** 2026-09-09

**Authors:** Reyyan Gökçen İşcan, Müşerref Banu Yılmaz, Büşra Bucak, Önder Tosun, Mine Güray Uzun, Sultan Seren Karakuş, Pınar Kumru

**Affiliations:** 1 Department of Gynecology and Obstetrics, Zeynep Kamil Women and Children Diseases Training and Research Hospital, University of Health Sciences, Istanbul 34668, Türkiye; 2 Department of Gynecologic Oncology, Zeynep Kamil Women and Children Diseases Training and Research Hospital, University of Health Sciences, Istanbul 34668, Türkiye

**Keywords:** refractory overactive bladder, idiopathic, onabotulinumtoxinA, urgency urinary incontinence, bladder trabeculation, treatment failure, retreatment

## Abstract

**Background/Objective**: Intradetrusor onabotulinumtoxinA is an established treatment for refractory idiopathic overactive bladder (OAB); however, data on predictors of treatment response and patient management after the initial injection are limited. This study evaluated the clinical outcomes, safety, factors associated with treatment failure, and retreatment pathways following intradetrusor onabotulinumtoxinA in women with refractory idiopathic OAB. **Methods**: This retrospective cohort study included 41 women with refractory idiopathic OAB treated with 100 U intradetrusor onabotulinumtoxinA between November 2022 and January 2026. Efficacy was assessed using 3-day bladder diaries, validated patient-reported outcome measures (UDI-6, IIQ-7, and OAB-V8), and the Treatment Benefit Scale (TBS). Safety outcomes, factors associated with treatment failure, retreatment preferences, and subsequent management strategies were analyzed. **Results**: Significant improvements were observed in daytime frequency, urgency episodes, urgency urinary incontinence episodes, and validated patient-reported outcome measures (all *p* < 0.001). Treatment success according to the TBS was achieved in 68.3% and 46.3% of patients at 12 and 24 weeks, respectively. Diffuse bladder trabeculation was independently associated with treatment failure at 12 weeks (aOR 11.90, 95% CI 1.99–71.14; *p* = 0.007), whereas no significant independent association was observed at 24 weeks. Urinary tract infection occurred in 19.5% of patients, and temporary clean intermittent catheterization was required in one patient (2.4%). By postoperative week 24, 53.7% of patients declined repeat treatment, most commonly because of inadequate symptom improvement (77.3%). Among those declining reinjection, 59.1% resumed or continued pharmacological therapy. **Conclusions**: Intradetrusor injection of 100 U onabotulinumtoxinA was associated with significant improvement and acceptable safety in women with refractory idiopathic OAB. Diffuse bladder trabeculation may help identify patients at risk of poor treatment response, warranting further validation in larger patient cohorts. Documenting retreatment decisions and management provides real-world evidence that may support long-term counseling and decision-making.

## 1. Introduction

Overactive bladder (OAB) is a symptom-defined syndrome characterized by urinary urgency, usually accompanied by increased daytime frequency and nocturia, with or without urgency urinary incontinence (UUI), in the absence of urinary tract infection or other identifiable pathology, as defined by the International Continence Society (ICS) [1]. OAB is a common condition worldwide, with a recent systematic review and meta-analysis reporting an overall prevalence of 19.5%, which increases with age and occurs more frequently in women than in men [2]. OAB affects more than just physical health; it also significantly reduces the quality of life, social engagement, emotional well-being, and work performance. Individuals with urgency urinary incontinence frequently experience feelings of embarrassment, limit their activities, and withdraw from daily and social interactions, leading to a substantial decline in their overall quality of life [3].

Behavioral and lifestyle approaches, such as bladder training, pelvic floor muscle exercises, fluid and diet adjustments, increased physical activity, and weight management, are essential for managing OAB [4,5]. When these conservative strategies do not sufficiently relieve symptoms, pharmacological treatments such as antimuscarinic agents or β3-adrenoceptor agonists are recommended [4,5,6]. Long-term persistence with pharmacological therapy for OAB remains poor, as many patients discontinue treatment because of inadequate symptom relief, adverse effects, treatment burden, or unmet treatment expectations, leaving a substantial proportion of patients with persistent symptoms [7,8]. Therefore, the current AUA/SUFU guidelines advocate a shared decision-making approach, allowing treatment to be individualized according to symptom severity, patient preferences, and clinical characteristics while facilitating timely escalation to minimally invasive therapies when appropriate [6].

For patients who remain symptomatic despite conservative and pharmacological therapies, intradetrusor onabotulinumtoxinA injection has become an established minimally invasive treatment option and is recommended by international guidelines [4,5,6]. OnabotulinumtoxinA reduces detrusor overactivity and improves urgency-related symptoms by inhibiting acetylcholine release and modulating bladder-afferent signaling. Randomized controlled trials and observational cohort studies have consistently demonstrated significant improvements in urinary urgency, UUI, daytime frequency, patient-reported outcomes, and health-related quality of life after treatment [3,9,10,11]. Among the available dosing regimens, 100 U is generally regarded as providing the most favorable balance between efficacy and safety in patients with idiopathic OAB [12]. However, treatment-related adverse events, including increased post-void residual volume, urinary tract infection, and the potential need for intermittent catheterization, should be considered during patient selection and counseling [11,12].

The efficacy and safety of intradetrusor onabotulinumtoxinA are well established, but little is known about how patients proceed after the initial treatment in routine clinical practice. Because the therapeutic effect is temporary and repeat injections are often required to maintain symptom control, patient attitudes toward retreatment may play an important role in long-term success.

Therefore, this study aimed to evaluate the real-world clinical outcomes of onabotulinumtoxinA in women with refractory idiopathic OAB and identify factors associated with treatment failure, patients’ decisions regarding repeat injections, and subsequent management following treatment discontinuation.

## 2. Materials and Methods

### 2.1. Ethical Approval

The study was approved by the Institutional Review Board of Zeynep Kamil Women and Children Diseases Training and Research Hospital (approval date: 23 July 2025; approval number: 92). All procedures were performed in accordance with the principles of the Declaration of Helsinki and its subsequent amendments. Written informed consent was obtained from all patients prior to receiving OnabotulinumtoxinA injections. The requirement for additional informed consent for the retrospective analysis of anonymized data was waived by the Institutional Review Board.

### 2.2. Study Design and Patient Population

This retrospective cohort study evaluated the efficacy, safety, factors associated with treatment outcomes, and retreatment preferences following intradetrusor onabotulinumtoxinA injections in women with refractory idiopathic overactive bladder (OAB) treated at a tertiary referral hospital between November 2022 and January 2026.

Women aged ≥18 years with a diagnosis of idiopathic OAB who had an inadequate response to conservative treatment and at least two anticholinergic and/or β3-adrenergic agonist (mirabegron) and who were scheduled to receive intradetrusor onabotulinumtoxinA for the first time were included. Patients who discontinued medical therapy because of adverse effects were also included in the study. All patients were informed about the potential need for clean intermittent catheterization (CIC) following treatment and agreed to undergo CIC if required.

Before treatment, all patients underwent clinical evaluation, completed a 3-day voiding diary, and underwent urodynamic assessments. Patients with neurogenic lower urinary tract dysfunction, bladder outlet obstruction, maximum urinary flow rate < 15 mL/s with an obstructive pattern, post-void residual urine volume (PVR) ≥ 100 mL at two separate measurement time points, recurrent urinary tract infection, gross hematuria, or stress-predominant mixed urinary incontinence were excluded.

### 2.3. Botulinum Toxin Injection Technique

Urinalysis and urine culture were obtained before treatment, and active urinary tract infection was excluded in all patients. Antibiotic prophylaxis was administered using 100 mg oral nitrofurantoin for 7 days following the procedure, in accordance with the institutional practice.

Injections were performed in the lithotomy position under local or regional anesthesia or sedation. For local anesthesia, 25 mL of 2% intravesical lidocaine was instilled and retained for 20–30 min before the procedure.

A total dose of 100 U onabotulinumtoxinA (Botox^®^, Allergan, Irvine, CA, USA) was diluted in 10 mL of normal saline and injected cystoscopically. All patients received a total of 10 intradetrusor injections (1 mL per injection), with the total dose (100 U) and injection volume (10 mL) remaining identical across patients. In patients whose urgency episodes consistently resulted in UUI, one of the 10 injection sites was placed in the trigone when the surgeon considered the trigonal injection technically safe and appropriate based on the intraoperative assessment. In the remaining patients, all the injection sites were located outside the trigone. Thus, trigonal injection represented the replacement of one of the 10 injection sites rather than an additional injection. All injections were administered at an approximate depth of 5 mm into the detrusor muscle using a rigid cystoscope by a gynecologist with experience in urogynecology.

Diffuse bladder trabeculation was assessed and documented during cystoscopy. This finding was defined as trabeculation involving at least half of the bladder’s surface.

Patients without macroscopic hematuria were discharged on the day of the procedure, whereas those receiving regional anesthesia were discharged after routine postoperative monitoring.

### 2.4. Follow-Up and Outcome Measures

The patients were evaluated at postoperative weeks 2, 12, and 24. All patients completed the 24-week follow-up assessment and were included in the final analyses.

The 2-week follow-up visit focused primarily on safety assessments; however, adverse events (AEs) were monitored and recorded throughout the study period at any unscheduled visit prompted by the patient’s symptoms. AEs occurring within 12 weeks of intradetrusor onabotulinumtoxinA injection were considered potentially treatment-related. Urinalysis was performed in all patients, and urine culture was obtained when clinically indicated. Postoperative urinary tract infection (UTI) was defined as bacterial growth >10^5^ CFU/mL on urine culture within 12 weeks after treatment, irrespective of the presence of urinary symptoms.

Preoperative PVR was measured by urethral catheterization in all patients. At the postoperative visit, PVR was screened using ultrasonography and subsequently confirmed by urethral catheterization when the PVR value obtained by ultrasound was ≥100 mL.

Patients with symptoms of voiding dysfunction and/or PVR ≥ 100 mL underwent additional clinical evaluation. Temporary CIC was recommended for patients with PVR ≥ 350 mL, regardless of symptoms, or for those with PVR between 200 and 349 mL and clinically significant voiding symptoms (e.g., difficulty voiding or a sensation of incomplete bladder emptying). This recommendation is consistent with the criteria used in two large phase 3 randomized controlled studies of 100 U onabotulinumtoxinA [3,10].

Treatment efficacy was assessed at postoperative week 12 using a 3-day voiding diary and patient-reported outcome measures, including the Urogenital Distress Inventory-6 (UDI-6), Incontinence Impact Questionnaire-7 (IIQ-7), and Overactive Bladder Questionnaire-V8 (OAB-V8). Validated Turkish-language versions of the UDI-6, IIQ-7, and OAB-V8 were used [13,14]. The UDI-6 consists of six items scored from 0 to 3, yielding a total score ranging from 0 to 18, whereas the IIQ-7 consists of seven items scored from 0 to 3, yielding a total score ranging from 0 to 21. Higher scores indicate greater symptom distress and impact on quality of life. The OAB-V8 consists of eight items scored from 0 to 5, yielding a total score ranging from 0 to 40, with higher scores indicating a greater OAB symptom burden. For each questionnaire, the total score was calculated by summing individual item scores. The evaluated diary parameters included daily frequency, urgency episodes, nocturia episodes, and urgency urinary incontinence episodes.

Subjective treatment success was assessed at postoperative weeks 12 and 24 using the Treatment Benefit Scale (TBS). Patients rated their condition as “greatly improved,” “improved,” “unchanged,” or “worsened” compared with their pretreatment status. Consistent with previous validation studies, “greatly improved” and “improved” responses were classified as treatment success, whereas “unchanged” and “worsened” responses were classified as treatment failure [15].

At the 24-week follow-up, patients were asked about their willingness to undergo repeat intradetrusor botulinum toxin injections. For patients who declined retreatment, the primary reasons were recorded as insufficient benefit from the initial injection, reluctance to undergo another invasive procedure, or inability to attend follow-up visits.

Repeat onabotulinumtoxinA injections were performed only after the 24-week TBS assessment was completed. Subsequent management strategies after refusal of retreatment were documented.

*The primary outcomes* were changes in bladder diary parameters, patient-reported outcome measures (OAB-V8, UDI-6, and IIQ-7), and subjective treatment success assessed using the TBS at 12 weeks postoperatively. *Secondary outcomes* included factors associated with treatment failure, safety outcomes, TBS findings, and retreatment preferences at postoperative week 24, and subsequent management strategies following retreatment refusal.

The onset and duration of the treatment effect were recorded based on patient reports. Duration was calculated from the initial onabotulinumtoxinA injection to the initiation of any additional non-conservative treatment for recurrent OAB symptoms, including pharmacological therapy, transcutaneous tibial nerve stimulation (TTNS), or repeat onabotulinumtoxinA injection. Patients who continued to experience treatment benefits without requiring additional non-conservative treatment at their last follow-up were considered to have an ongoing treatment effect.

### 2.5. Statistical Analysis

Statistical analyses were performed using IBM SPSS Statistics for Windows version 21.0 (IBM Corp., Armonk, NY, USA). Continuous variables are presented as mean ± standard deviation or median (minimum–maximum), depending on the data distribution, and categorical variables are presented as frequencies and percentages.

Normality was assessed using the Shapiro–Wilk test. Comparisons between baseline and follow-up measurements were performed using the paired *t*-test or Wilcoxon signed-rank test, as appropriate. For comparisons performed with the Wilcoxon signed-rank test, the magnitude of change was additionally estimated using the Hodges–Lehmann estimator with 95% confidence intervals (CIs). Categorical variables were compared using the chi-square or Fisher’s exact test, as appropriate.

To identify factors associated with treatment outcomes, univariate analyses were first performed for demographic, clinical, urodynamic, and procedure-related variables. Separate multivariable logistic regression models were constructed for treatment failure at 12 and 24 weeks, with results reported as adjusted odds ratios (aORs), 95% CIs, and *p*-values.

Given the small sample size and limited number of treatment failure events, Firth penalized logistic regression was additionally performed as a sensitivity analysis to assess the potential small-sample and sparse-data bias and the robustness of the findings. This analysis was conducted using R software version 4.6.1 (R Foundation for Statistical Computing, Vienna, Austria) with the *logistf* package. A two-sided *p*-value < 0.05 was considered statistically significant.

## 3. Results

### 3.1. Patient Characteristics

A total of 41 women with refractory overactive bladder underwent intradetrusor onabotulinumtoxinA injection. The baseline demographic and clinical characteristics of the study population are shown in Table 1. The patients had a mean age of 63.2 ± 10.7 years and a mean BMI of 32.5 ± 6.4 kg/m^2^, reflecting an overall obese population. Mixed urinary incontinence was identified in 25 patients (61.0%), and 16 patients (39.0%) had undergone previous urogynecological surgery. The median duration of urinary incontinence prior to treatment was 5 years (range, 1–17). Before receiving intradetrusor botulinum toxin injections, the patients were treated with antimuscarinic agents and/or β3-adrenergic agonist for a median duration of 12 months.

### 3.2. Efficacy Outcomes

Significant improvements were observed in both bladder diary parameters and patient-reported outcome measures after intravesical onabotulinumtoxinA treatment (Table 2). The mean daily frequency, urgency episodes, and UUI episodes decreased significantly after treatment (all *p* < 0.001). Similarly, the UDI-6, IIQ-7, and OAB-V8 scores showed substantial improvement. Although the number of nocturia episodes decreased, the difference was not statistically significant (*p* = 0.057).

**Figure 1:**
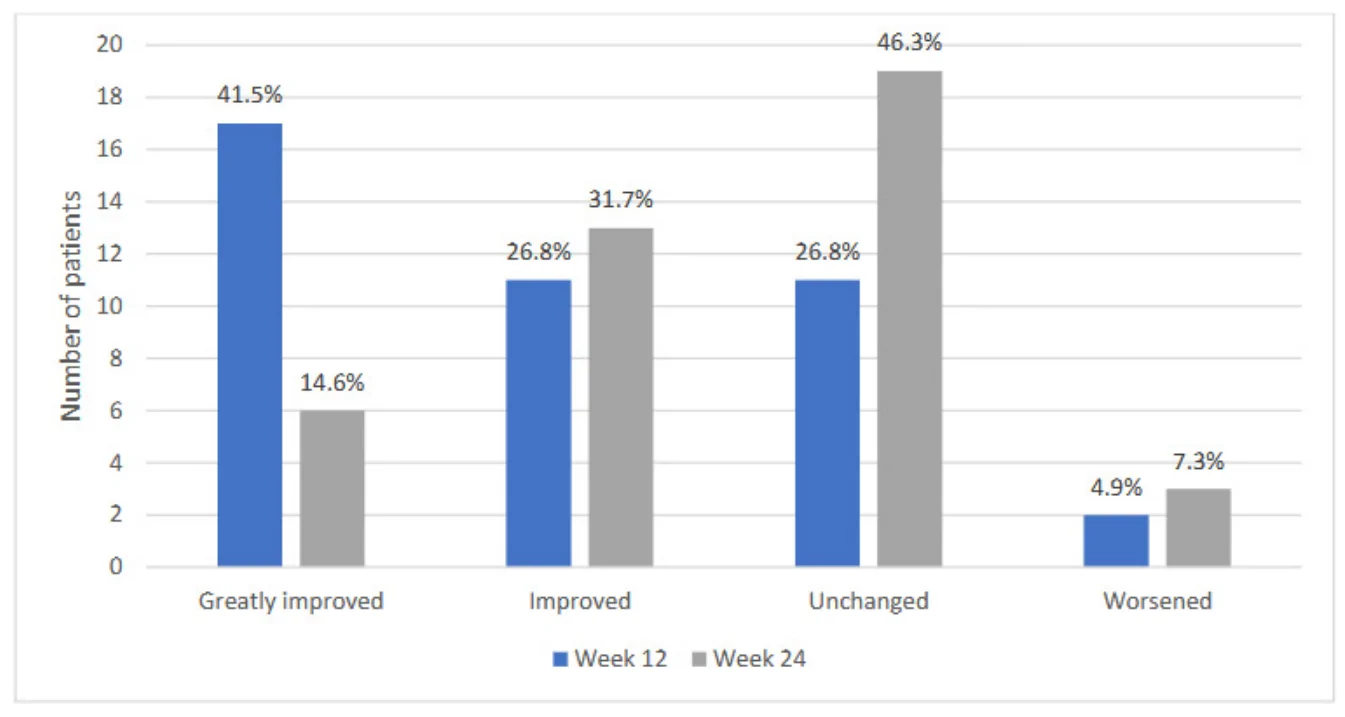
Distribution of Treatment Benefit Scale (TBS) responses at postoperative weeks 12 and 24, following intradetrusor onabotulinumtoxinA injection.

The responses to the Treatment Benefit Scale (TBS) are shown in Figure 1. At postoperative week 12, 28 patients (68.3%) were classified as treatment successes, while 13 (31.7%) were classified as treatment failures. By postoperative week 24, the proportion of patients demonstrating improvement decreased to 46.3% (*n* = 19), with 22 patients (53.7%) classified as having treatment failure.

Patients rated their overall bladder condition compared with the baseline using the Treatment Benefit Scale and classified their responses as greatly improved, improved, unchanged, or worsened. The data labels indicate the corresponding numbers and percentages of patients in each response category.

### 3.3. Predictors of Treatment Failure

Several demographic and clinical variables, including cystoscopic and urodynamic findings, were evaluated for their association with treatment failure at postoperative weeks 12 and 24 (Table 3). Trigonal injection was performed in 18 patients (43.9%) and was not significantly associated with treatment failure at either time point.

At 12 weeks, diffuse bladder trabeculation was significantly more common in the treatment failure group than in the treatment success group (69.2% vs. 17.9%, *p* = 0.003). No significant associations were identified between treatment outcomes and demographic characteristics, comorbidities, previous urogynecological surgery, mixed urinary incontinence, duration of prior medical therapy, or other preoperative urodynamic findings.

At 24 weeks, low bladder compliance was significantly more frequent in the treatment failure group than in the treatment success group (72.7% vs. 36.8%, *p* = 0.030). Diffuse bladder trabeculation was also significantly associated with treatment failure (50.0% vs. 15.8%, *p* = 0.046).

Separate multivariable logistic regression models were performed for treatment failure at 12 and 24 weeks (Table 4). At 12 weeks, diffuse bladder trabeculation was independently associated with treatment failure (aOR 11.90, 95% CI 1.99–71.14; *p* = 0.007), whereas low bladder compliance was not (aOR 0.75, 95% CI 0.12–4.66; *p* = 0.756). At 24 weeks, neither diffuse bladder trabeculation (adjusted odds ratio [aOR] 3.46, 95% confidence interval [CI] 0.70–17.16; *p* = 0.128) nor low bladder compliance (aOR 3.01, 95% CI 0.72–12.56; *p* = 0.132) was independently associated with treatment failure.

The Firth penalized logistic regression sensitivity analysis yielded results consistent with the primary analysis. The association between diffuse bladder trabeculation and treatment failure at 12 weeks remained statistically significant (OR 9.17, 95% CI 1.99–57.09; *p* = 0.004), whereas no significant independent associations were observed for trabeculation or low bladder compliance at 24 weeks.

### 3.4. Safety and Follow-Up Outcomes

All patients completed the 24-week assessment, and long-term follow-up data were available for a median of 12 months (range, 6–42 months). Most patients reported noticing a treatment effect within the first postoperative week (70.7%, *n* = 29), while 26.8% (*n* = 11) first noted improvement during the second week, and one patient (2.4%) during the fourth week.

Postoperative urinary tract infections occurred in eight patients (19.5%), with no recurrent infections observed during the postoperative follow-up period and were successfully treated with antibiotic therapy. All eight patients who developed postoperative UTI had a history of urogynecological surgery. Among the 16 patients with previous urogynecological surgery, UTI occurred in 8 (50.0%), whereas no UTI was observed among the 25 patients without such a history (0%). This difference was statistically significant (Fisher’s exact test, *p* < 0.01).

Postvoid residual volume increased from 24.1 ± 31.2 mL [median, 0 (0–90)] at baseline to 45.1 ± 54.5 mL [median, 40 (0–225)] at postoperative week 2 (*p* = 0.03). Four patients developed elevated postoperative PVR (≥100 mL); however, only one patient (2.4%) required temporary CIC. All cases of elevated postoperative PVR were resolved during follow-up.

A 77-year-old woman experienced generalized weakness during the second postoperative week that gradually resolved over 4 months. Another patient developed macroscopic hematuria after the second injection while continuing acetylsalicylic acid therapy, which resolved after two days of bladder irrigation.

Among patients classified as treatment successes at postoperative week 12, the median duration of the treatment effect was 6 months (min–max; 3–29 months), with a mean duration of 7.7 ± 5.2 months. Treatment benefit persisted for ≥12 months in four patients. Of these, two subsequently underwent repeat onabotulinumtoxinA injection, while one patient received TTNS. In the patient with the longest treatment-effect duration, daytime urgency and urinary incontinence remained absent despite the recurrence of nocturnal urgency from month 10; TTNS was initiated at month 29 following the recurrence of urinary incontinence.

Stress urinary incontinence (SUI) symptoms persisted in 15 of 25 patients (60.0%) with mixed urinary incontinence at 12 weeks postoperatively.

### 3.5. Retreatment Preferences and Subsequent Management

Following completion of the 24-week TBS assessment, patients were surveyed regarding their willingness to undergo repeat intradetrusor onabotulinumtoxinA treatment. No repeat injections were administered before this assessment. Overall, 53.7% (*n* = 22) declined further injection. Willingness to undergo retreatment was significantly higher among patients with treatment success at 24 weeks than among those with treatment failure (63.2% [12/19] vs. 31.8% [7/22], respectively; χ^2^ = 4.03, *p* = 0.045). Treatment success rates did not differ significantly between patients with pure UUI and those with MUI at either 12 weeks (*p* = 0.524) or 24 weeks (*p* = 0.707). Similarly, retreatment willingness did not differ significantly between the two groups (56.3% [9/16] vs. 40.0% [10/25], respectively; χ^2^ = 1.04, *p* = 0.309). However, among patients with MUI, retreatment willingness was significantly lower in those with persistent postoperative SUI than in those without persistent SUI (13.3% [2/15] vs. 80.0% [8/10], respectively; Fisher’s exact *p* = 0.002).

The primary reason cited for declining repeat treatment was inadequate benefit from the initial injection (77.3%, *n* = 17), followed by reluctance to undergo another invasive procedure (13.6%, *n* = 3) and inability to attend follow-up appointments (9.1%, *n* = 2).

**Figure 2:**
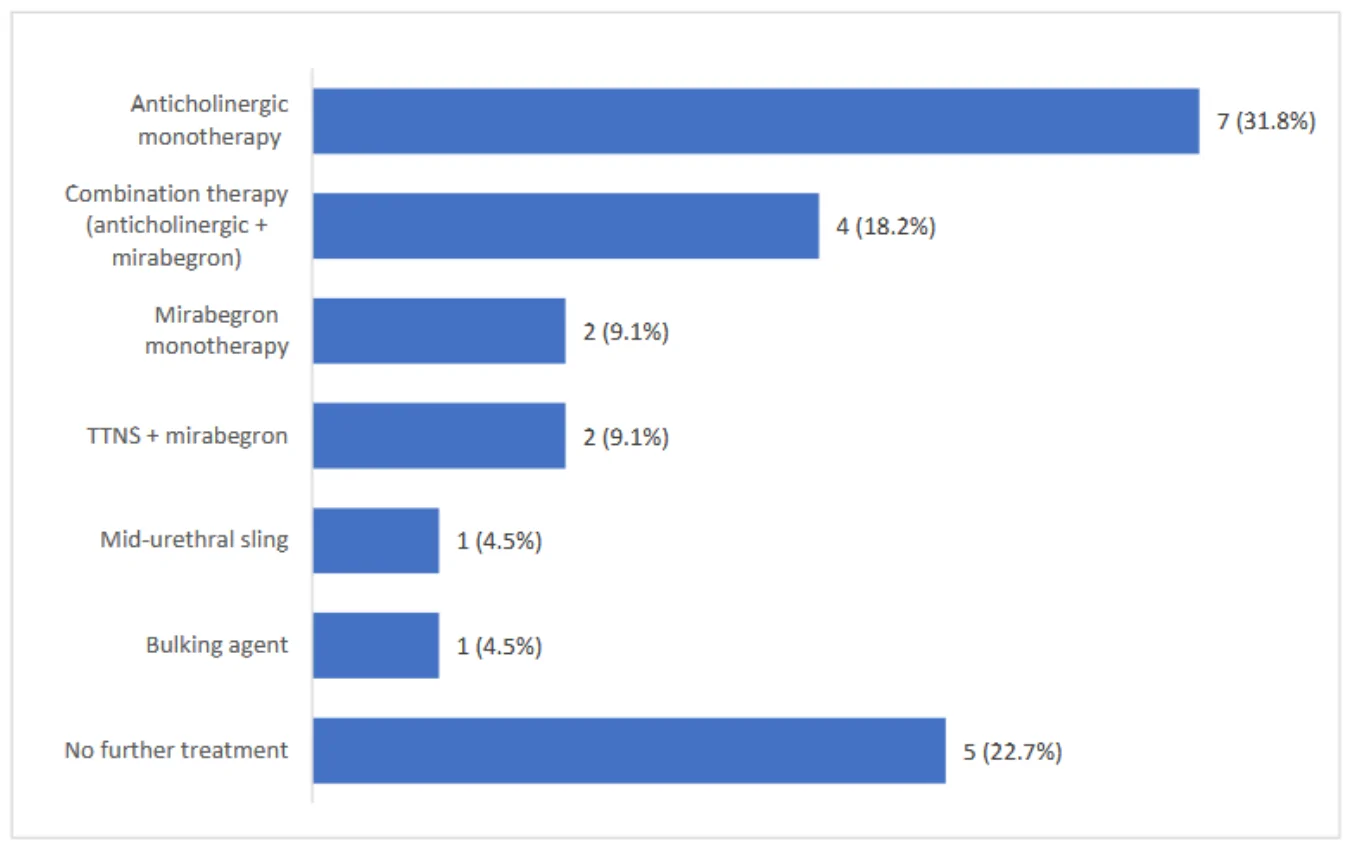
Subsequent management strategies for patients who declined repeat intradetrusor onabotulinumtoxinA injection.

Among the 22 patients who refused retreatment, seven were classified as treatment successes at 24 weeks. Of these, five presented with mixed urinary incontinence, characterized by persistent stress urinary incontinence despite improvement in urgency symptoms. The remaining two patients, both with pure urgency urinary incontinence, declined further injections despite symptomatic improvement, primarily due to their reluctance to undergo additional invasive procedures. The subsequent management strategies for patients who refuse retreatment are shown in Figure 2.

Data are presented as the number and percentage of patients in each management category among those who declined repeat intradetrusor onabotulinumtoxinA injections (*n* = 22); TTNS: transcutaneous tibial nerve stimulation.

Among the patients willing to undergo repeat treatment, seven proceeded with additional injections (with a maximum of five injections in a single patient), eight had not yet received repeat injections at their most recent follow-up, three were lost to follow-up after the 24-week assessment, and one patient with mixed urinary incontinence opted for a mid-urethral sling procedure instead.

## 4. Discussion

In this cohort of women with refractory idiopathic overactive bladder, intradetrusor onabotulinumtoxinA was associated with significant improvements in urinary symptoms, patient-reported outcomes, and subjective treatment success while maintaining an acceptable safety profile. Diffuse bladder trabeculation was independently associated with treatment failure at 12 weeks after multivariable adjustment, highlighting a potentially important and relatively unexplored factor associated with treatment response. We also evaluated retreatment preferences and subsequent management after the initial injection, providing clinical data on aspects that remain insufficiently explored in the literature.

In the present study, treatment with intradetrusor onabotulinumtoxinA resulted in significant improvements in both bladder diary parameters and validated patient-reported outcome measures. These findings align with evidence supporting the clinical benefits of intradetrusor onabotulinumtoxinA in patients with refractory idiopathic OAB [3,6,9,10,11,16,17,18]. More than two-thirds of patients reported treatment success according to the TBS at postoperative week 12. Approximately 70% of patients reported the onset of clinical benefits within the first postoperative week, consistent with the findings of a prospective multinational observational study involving 504 patients [19]. The mean duration of the treatment effect was 7.7 months, similar to that observed in the long-term extension study of 100 U onabotulinumtoxinA [20]. As expected, the proportion of patients reporting treatment success declined from 68.3% at postoperative week 12 to 46.3% at week 24, reflecting the temporary nature of the treatment effect of onabotulinumtoxinA.

The adverse event profile in our cohort was consistent with previous reports, with urinary tract infection (UTI) being the most frequent adverse event, affecting 19.5% of patients. All patients with postoperative UTI had prior urogynecological surgery for pelvic organ prolapse or SUI. UTI occurred significantly more often in these patients than in those without such history (50.0% vs. 0%; Fisher’s exact test, *p* < 0.01), indicating a potential link. However, UTI rates may also depend on peri-procedural factors like antibiotic prophylaxis, which varies widely. The optimal antibiotic choice and duration for intradetrusor onabotulinumtoxinA injections remain unclear [21].

The need for temporary CIC in our cohort (2.4%) was lower than that reported in previous 100 U onabotulinumtoxinA studies [3,10,17]. This may be partly explained by the relatively small size of our cohort and the inclusion of only women, as men have been reported to experience higher rates of incomplete bladder emptying requiring catheterization following botulinum toxin administration [22]. The only patient who required temporary CIC also had a history of pelvic organ prolapse surgery and mid-urethral sling, with documented episodes of elevated PVR before onabotulinumtoxinA treatment despite a normal baseline PVR at preprocedural evaluation. Although these observations are based on a limited number of patients, we suggest that previous urogynecological surgery warrants further investigation as a potential factor associated with UTIs, voiding dysfunction, and the need for temporary CIC after botulinum toxin injections. Two uncommon adverse events were also observed. One patient experienced transient generalized weakness during the second postoperative week, which gradually resolved during follow-up. Although generalized weakness has been reported after botulinum toxin exposure, systemic adverse effects are uncommon following lower urinary tract administration of 100 U onabotulinumtoxinA and have predominantly been described after higher-dose treatment [23]. Therefore, it remains uncertain whether this event is directly related to the treatment. Another patient developed macroscopic hematuria, which was considered more likely attributable to continued acetylsalicylic acid use than to the intradetrusor injection itself.

Identifying predictors of treatment failure for intravesical botulinum toxin may facilitate patient selection and improve preoperative counseling. However, most studies evaluating factors associated with treatment outcomes have included both sexes, whereas evidence from women-only cohorts is limited [24,25]. A recent prospective study involving 94 women with idiopathic OAB evaluated the clinical outcomes in a predominantly older and overweight cohort [25]. However, only three patients experienced poor outcomes, precluding a meaningful assessment of the factors associated with failure. Similarly, our study included women with idiopathic OAB, representing a cohort with a mean BMI within the obese range. Categorizing patients into treatment success and failure groups according to TBS, we performed univariate analyses of variables associated with efficacy. Diffuse bladder trabeculation was associated with failure at 12 and 24 weeks, while low bladder compliance was associated with failure at 24 weeks. In multivariable analysis, diffuse bladder trabeculation remained independently associated with failure at 12 weeks, whereas this association was no longer significant at 24 weeks. Low bladder compliance did not remain associated with failure at 24 weeks. Firth penalized logistic regression sensitivity analysis was consistent with multivariable analysis, supporting the association between diffuse bladder trabeculation and failure at 12 weeks. Given the small sample size, limited number of patients with diffuse trabeculation, and wide confidence intervals, this association should be considered hypothesis-generating rather than evidence of an established independent predictor and requires confirmation in larger cohorts. Bladder trabeculation is a cystoscopic sign of long-standing bladder remodeling caused by persistent mechanical and/or functional stress. This process is characterized by detrusor hypertrophy, followed by extracellular matrix changes and increased collagen deposition, which may alter the biomechanical properties of the bladder wall and contribute to dysfunction [26]. Trabeculation may therefore reflect chronic structural and functional changes rather than directly determine treatment response. Although traditionally associated with bladder outlet obstruction, studies in women have also linked trabeculation to detrusor overactivity and UUI, suggesting a shared pathophysiological pathway involving detrusor hypertrophy and involuntary contractions [27,28]. To our knowledge, only one prospective study has examined its association with response to intradetrusor botulinum toxin injection in women with refractory idiopathic OAB [29]. However, that study evaluated abobotulinumtoxinA and administered higher doses to patients with moderate-to-severe trabeculation, making it difficult to isolate the effect of trabeculation itself. In contrast, all patients in our study received a uniform dose of 100 U of onabotulinumtoxinA, reducing this potential source of bias.

Other mechanisms may also contribute to treatment failure. Neutralizing antibody (NAb) formation may reduce the pharmacological activity and clinical efficacy of onabotulinumtoxinA, although its occurrence and clinical impact appear limited [30]. In a recent meta-analysis, post-treatment NAbs were detected in only three of 956 patients (0.3%) treated for OAB, with no cases of secondary non-response attributed to NAb formation. All patients in our cohort were undergoing intradetrusor onabotulinumtoxinA treatment for the first time, and NAbs were not assessed; therefore, their potential contribution to treatment failure could not be directly evaluated.

Previous long-term studies have offered important insights into how long patients continue treatment and why some stop receiving repeat intradetrusor onabotulinumtoxinA injections [20,31,32]. Building on this, our study provides further details on the subsequent management of patients who declined reinjection in a homogeneous cohort of women with refractory, idiopathic OAB. One of the most notable findings was that more than half of the patients (22/41, 53.7%) did not wish to undergo repeat injections by week 24. This proportion was substantially higher than that reported in previous studies, including the study by Dowson et al., in which only approximately one-quarter of patients declined repeat treatment despite receiving a 200-U dose of onabotulinumtoxinA [33]. Notably, none of the patients in our study cited adverse events as the primary reason for refusing to reinject. Instead, inadequate treatment benefit was the most frequently reported reason (17/22, 77.3%). Importantly, retreatment decisions did not fully correspond to treatment response, as seven of the 22 patients who declined reinjection were nevertheless classified as treatment successes at 24 weeks. Additional analyses showed that, among patients with MUI, retreatment willingness was markedly lower in those with persistent postoperative SUI than in those without persistent SUI (13.3% vs. 80.0%). These findings suggest that persistent SUI may influence patients’ overall perception of treatment benefit and retreatment decisions even when the OAB component has responded favorably.

It is noteworthy that a significant proportion of patients (13/22, 59.1%) who refused reinjection chose to either resume or maintain their pharmacological treatment. This finding should be interpreted in light of local treatment availability, as sacral neuromodulation and percutaneous tibial nerve stimulation were not available at our hospital. Nevertheless, these management patterns may reflect routine practice in centers where access to the full range of third-line OAB therapies is limited.

Our study had some limitations. The relatively small sample size, particularly the small number of patients with diffuse bladder trabeculation, limited the multivariable analyses and contributed to the wide confidence intervals around the effect estimates. The association between diffuse trabeculation and treatment failure should therefore be interpreted with caution. To mitigate potential small-sample and sparse-data bias, we performed a Firth penalized logistic regression as a sensitivity analysis, which yielded findings consistent with the standard multivariable analysis. The retrospective nature of the study is another limitation and may have introduced selection or information bias; however, the use of predefined eligibility criteria and a relatively homogeneous cohort of women with refractory idiopathic OAB may have reduced this risk. Bladder trabeculation was defined as involvement of at least half of the bladder surface, but its assessment was based on visual inspection during cystoscopy. Given the lack of a standardized grading system for bladder trabeculation, particularly in women, some degree of misclassification remains possible. Moreover, although the total dose, injection volume, and number of injection sites were standardized across all patients, the inclusion of the trigone in selected patients introduced some procedural heterogeneity. Finally, the single-center design may limit the generalizability of our findings to other settings.

Despite these limitations, this study has notable strengths. Importantly, our study specifically evaluated the association between diffuse bladder trabeculation and treatment response in a women-only cohort receiving a uniform 100-U dose of onabotulinumtoxinA, addressing a clinically relevant relationship that remains scarcely investigated in the literature. All intradetrusor injections were performed using a standardized technique by an experienced gynecologist to minimize the procedural variability. Complete 24-week follow-up data were available for all patients, and more than half of the participants (24/41, 58.5%) were monitored for at least 12 months. We also systematically documented patients’ reasons for declining repeat treatment and their subsequent management strategies, providing real-world clinical information that has received limited attention in previous long-term studies. Furthermore, the inclusion of a homogeneous cohort of women with refractory idiopathic OAB reduced clinical variability and enhanced the internal validity of our findings.

## 5. Conclusions

OnabotulinumtoxinA significantly improved daytime frequency, urgency, and UUI episodes, according to bladder diary outcomes, as well as validated patient-reported outcome measures and subjective treatment benefits in women with refractory idiopathic OAB. Our findings suggest that diffuse bladder trabeculation may help identify patients who are less likely to achieve a favorable response to treatment, although this finding requires confirmation in larger prospective cohorts. By documenting patients’ reasons for declining repeat injections and their subsequent treatment pathways, our study provides real-world insights into clinical decision-making beyond the initial treatment response. These findings may inform the long-term management of patients discontinuing botulinum toxin therapy and contribute to a better understanding of the factors influencing retreatment decisions.

## Figures and Tables

**Table 1:** Baseline demographic and clinical characteristics of patients with refractory overactive bladder undergoing intradetrusor onabotulinumtoxinA treatment (*n* = 41).

Variable	
Age (years), mean ± SD	63.2 ± 10.7
BMI (kg/m^2^), mean ± SD	32.5 ± 6.4
Gravida, median (min–max)	4.0 (0–7)
Parity, median (min–max)	3.0 (0–6)
Diabetes mellitus, *n* (%)	17 (41.5)
Hypertension, *n* (%)	25 (61.0)
Thyroid disease *n* (%)	8 (19.5)
History of previous urogynecological surgery, *n* (%)	16 (39.0)
Mixed urinary incontinence, *n* (%)	25 (61.0)
Duration of urinary incontinence (years), median (min–max)	5.0 (1.0–17.0)
Duration of prior antimuscarinic and/or β3 adrenergic agonist therapy (months), median (min–max)	12.0 (3.0–96.0)

BMI, body mass index; SD, standard deviation; *n*, number of patients.

**Table 2:** Changes in 3-day bladder diary parameters, symptom scores following intradetrusor onabotulinumtoxinA injection.

Variable	Time Point	Mean ± SD	Median (Min–Max)	Hodges–Lehmann Estimate of Change (95% CI)	*p*-Value
Mean frequency episodes/day	Baseline	9.0 ± 3.5	8 (4–17)	−2.20 (−3.15 to −1.35)	<0.001
	Postoperative Week 12	6.6 ± 3.2	6 (3–20)		
Mean urgency episodes/day	Baseline	8.8 ± 3.8	8 (3–21)	−4.00 (−5.30 to −2.60)	<0.001
	Postoperative Week 12	4.8 ± 3.9	4 (0–20)		
Mean nocturia episodes/night	Baseline	2.8 ± 1.7	3 (0–8.3)	−0.50 (−1.00 to 0.00)	0.057
	Postoperative Week 12	2.2 ± 1.5	2 (0–5)		
Mean urgency urinary incontinence episodes/day	Baseline	6.6 ± 3.5	5 (3–18.3)	−3.30 (−4.65 to −2.00)	<0.001
	Postoperative Week 12	3.1 ± 3.3	3 (0–15)		
UDI-6 total score	Baseline	11.8 ± 2.7	12 (6–18)	−4.00 (−5.50 to −3.00)	<0.001
	Postoperative Week 12	7.4 ± 4.0	6 (0–18)		
IIQ-7 total score	Baseline	14.4 ± 4.2	15 (4–21)	−6.00 (−8.00 to −4.00)	<0.001
	Postoperative Week 12	8.3 ± 4.8	7 (0–18)		
OAB-V8 total score	Baseline	26.4 ± 5.3	27 (10–38)	−10.50 (−14.00 to −7.50)	<0.001
	Postoperative Week 12	15.9 ± 8.2	15 (3–34)		

Wilcoxon signed-rank test. UDI-6, Urogenital Distress Inventory-6; IIQ-7, Incontinence Impact Questionnaire-7; OAB-V8, Overactive Bladder Questionnaire-V8.

**Table 3:** Univariate analysis of factors associated with treatment outcome at 12 and 24 weeks.

Variable	12-Week Success (*n* = 28)	12-Week Failure (*n* = 13)	*p* Value	24-Week Success (*n* = 19)	24-Week Failure (*n* = 22)	*p* Value
Medical treatment duration (months), median (min–max)	12 (3–96)	12 (4–60)	0.623	12 (3–33)	12 (3–96)	0.803
Pure UUI, *n* (%)	10 (35.7)	6 (46.2)	0.524	8 (42.1)	8 (36.4)	0.707
MUI, *n* (%)	18 (64.3)	7 (53.8)	0.732	11 (57.9)	14 (63.6)	0.757
Previous urogynecologic surgery, *n* (%)	9 (32.1)	7 (53.8)	0.302	8 (42.1)	8 (36.4)	0.757
Preoperative DO, *n* (%)	22 (78.6)	10 (76.9)	1.000	15 (78.9)	17 (77.3)	1.000
Low compliance, *n* (%)	14 (50.0)	9 (69.2)	0.321	7 (36.8)	16 (72.7)	**0.030**
Urodynamic SUI, *n* (%)	9 (32.1)	8 (61.5)	0.098	7 (36.8)	10 (45.5)	0.752
Reduced bladder capacity, *n* (%)	12 (42.9)	6 (46.2)	1.000	6 (31.6)	12 (54.5)	0.209
Trigonal injection, *n* (%)	11 (39.3)	7 (53.8)	0.503	7 (36.8)	11 (50.0)	0.531
Diffuse bladder trabeculation, *n* (%)	5 (17.9)	9 (69.2)	**0.003**	3 (15.8)	11 (50.0)	**0.046**

MUI, mixed urinary incontinence; DO, detrusor overactivity; SUI, stress urinary incontinence. Bold values indicate statistical significance (*p* < 0.05).

**Table 4:** Standard and Firth penalized multivariable logistic regression analyses of factors associated with treatment failure at 12 and 24 weeks.

	12-Week Standard aOR (95% CI)	*p* Value	12-Week Firth OR (95% CI)	*p* Value	24-Week Standard aOR (95% CI)	*p* Value	24-Week Firth OR (95% CI)	*p* Value
**Diffuse bladder trabeculation**	**11.90 (1.99–71.14)**	**0.007**	**9.17 (1.99–57.09)**	**0.004**	3.46 (0.70–17.16)	0.128	3.09 (0.71–15.37)	0.132
**Low bladder compliance**	0.75 (0.12–4.66)	0.756	0.82 (0.13–4.21)	0.818	3.01 (0.72–12.55)	0.132	2.80 (0.72–11.37)	0.137

aOR, adjusted odds ratio; OR, odds ratio; CI, confidence interval. **Note:** Treatment outcome was coded as 0 = success and 1 = failure. Absence of diffuse bladder trabeculation and normal bladder compliance were used as the reference categories (OR = 1.00). Firth penalized logistic regression was performed as a sensitivity analysis to account for potential small-sample and sparse-data bias. Bold values indicate statistical significance (*p* < 0.05).

## Data Availability

The datasets generated and analyzed during the current study are not publicly available due to patient confidentiality but are available from the corresponding author upon reasonable request.

## Abbreviations

AE	Adverse event(s)
BMI	Body mass index
CI	Confidence interval
CIC	Clean intermittent catheterization
DO	Detrusor overactivity
ICS	International Continence Society
IIQ-7	Incontinence Impact Questionnaire-7
OAB	Overactive bladder
OAB-V8	Overactive Bladder Questionnaire-V8
OR	Odds ratio
PVR	Post-void residual urine volume
SUI	Stress urinary incontinence
TBS	Treatment Benefit Scale
UDI-6	Urogenital Distress Inventory-6
UTI	Urinary tract infection
UUI	Urgency urinary incontinence
